# Association between the efficacy and immune-related adverse events of pembrolizumab and chemotherapy in non-small cell lung cancer patients: a retrospective study

**DOI:** 10.1186/s12885-022-10133-1

**Published:** 2022-10-06

**Authors:** Kana Kurokawa, Yoichiro Mitsuishi, Naoko Shimada, Yuta Kawakami, Keita Miura, Taichi Miyawaki, Tetsuhiko Asao, Ryo Ko, Takehito Shukuya, Rina Shibayama, Shuko Nojiri, Kazuhisa Takahashi

**Affiliations:** 1 Department of Respiratory Medicine, Juntendo University Faculty of Medicine and Graduate School of Medicine, 3-1-3 Hongo, Bunkyo-ku, Tokyo, 113-8431 Japan; 2 Department of Mathematics, Physics, Electrical Engineering and Computer Science, Graduate School of Engineering Science, Yokohama National University, 79-5 Hodogaya-ku, Tokiwadai, Kanagawa, 240-8501 Japan; 3 Medical Technology Innovation Center, Clinical Research and Trial Center, Juntendo University, 2-1-1 Hongo, Bunkyo-ku, Tokyo, 113-8421 Japan

**Keywords:** Non‐small cell lung cancer, Immune-checkpoint inhibitors, Combination therapy, Immune-related adverse events

## Abstract

**Background:**

The combination of immune-checkpoint inhibitors with chemotherapy has become the standard of treatment for non-small cell lung cancer (NSCLC) patients. However, the association between therapeutic efficacy and the development of immune-related adverse events (irAEs) remains unclear in patients treated with combination therapy. We aimed to investigate the frequency of irAEs, and the association between therapeutic efficacy and the development of irAEs in patients with NSCLC.

**Materials and methods:**

We retrospectively surveyed patients with chemo-naïve advanced NSCLC who received pembrolizumab plus platinum-based chemotherapy or pembrolizumab monotherapy at Juntendo University Hospital, Japan, between February 2017 and May 2021.

**Results:**

Among 148 patients (median [range] age, 68 (33–85) years; 107 men [72.3%] and 41 women [27.7%]), 74 each received pembrolizumab plus chemotherapy and pembrolizumab monotherapy. IrAEs were observed in 46 (62.2%) and 41 patients (55.4%) in the combination therapy and monotherapy group, respectively. Patients with irAEs showed significantly longer progression-free survival (PFS) than those without irAEs in the combination therapy group (8.9 vs. 5.7 months; Hazard Ratio [HR], 0.53; 95% CI, 0.29–0.98; *P* = 0.041) and monotherapy group (11.7 vs. 5.0 months; HR, 0.40; 95% CI, 0.22–0.70; *P* = 0.001). In the multivariable analysis, development of irAEs was positively associated with PFS in both the groups (HR, 0.48; 95% CI, 0.26–0.89; *P* = 0.019 and HR, 0.38; 95% CI, 0.21–0.68; *P* < 0.01). In the inverse probability of treatment weighting adjusted analysis, development of irAEs was significantly associated with combination therapy (OR, 0.56; 95% CI, 0.34–0.91; *P* = 0.019).

**Conclusion:**

Our study demonstrated that the incidence of irAEs was associated with favorable efficacy in patients treated with pembrolizumab plus chemotherapy, as well as pembrolizumab monotherapy. Also, the addition of chemotherapy to pembrolizumab significantly increased the incidence of irAEs.

**Supplementary Information:**

The online version contains supplementary material available at 10.1186/s12885-022-10133-1.

## Background

Immunotherapy has revolutionized the treatment of various types of cancers, including lung cancer [1]. Immune-checkpoint blockade agents inhibit pathways of the immune cascade, leading to an increase in the response against tumor cells [2]. For patients with advanced non-small cell lung cancer (NSCLC), monoclonal antibodies against the programmed cell death 1 (PD-1), programmed cell death ligand 1 (PD-L1), and cytotoxic T-lymphocyte-associated protein 4 have been approved in various treatment settings [3]. In addition to single-agent use, the effects of adding chemotherapy to immunotherapy have also been reported. For example, in the KEYNOTE-189 trial, the addition of pembrolizumab to chemotherapy resulted in significantly longer progression-free survival (PFS) and overall survival (OS) in patients with non-squamous NSCLC [4]. The KEYNOTE-407 trial showed similar results in patients with squamous NSCLC [5]. Furthermore, chemotherapy has been reported to promote tumor immunity by inducing immunogenic cell death and interfering with malignant cells’ strategies to evade immune recognition [6]. These effects might have contributed to favorable outcomes.

Despite the clinical benefits of immune checkpoint inhibitors (ICIs), checkpoint inhibition can induce a unique spectrum of side effects, known as immune-related adverse events (irAEs). IrAEs can involve any organ or system, such as the skin, gastrointestinal tract, and the lungs. They are generally mild but can sometimes have severe consequences and require hormone replacement therapy or immunosuppressants [7]. Some studies have shown that the development of irAEs is a predictive factor for ICI treatment efficacy in monotherapy [8]. However, it remains unknown whether the addition of chemotherapy to pembrolizumab changes the irAE profiles or the correlation between irAEs and prognosis in real-world settings.

We performed a retrospective study to investigate the frequency of irAEs, and the association between therapeutic efficacy and the development of irAEs in patients with advanced NSCLC either treated with pembrolizumab plus chemotherapy or pembrolizumab monotherapy.

## Methods

### Subject cohort and study design

All patients with NSCLC treated with pembrolizumab monotherapy or combined with platinum-based chemotherapy as first-line therapy at Juntendo University Hospital between February 2017 and May 2021 were eligible for this study. Patients were administered 200 mg of pembrolizumab monotherapy intravenously in 3-week cycles until disease progression was observed. In the combination therapy group, patients with non-squamous NSCLC were administered 200 mg of pembrolizumab, cisplatin (75 mg per square meter of body surface area) or carboplatin (area under the concentration–time curve, 5 mg per milliliter per minute), and pemetrexed (500 mg per square meter of body surface area). All drugs were administered intravenously every three weeks for four cycles, followed by pembrolizumab and pemetrexed every three weeks as maintenance therapy. Patients with squamous NSCLC received 200 mg of pembrolizumab and carboplatin (area under the concentration–time curve, 6 mg per milliliter per minute) on day 1 and either paclitaxel (200 mg per square meter of body-surface area) on day 1 or nab-paclitaxel (100 mg per square meter of body-surface area) on days 1, 8, and 15. All treatments were administered intravenously for four cycles in 3-week cycles, followed by pembrolizumab every three weeks. The dose and duration of drug administration were adjusted as per the clinician’s discretion.

The institutional review board of Juntendo University Hospital approved this study (Approval number H18-0083).

### Evaluation of patient characteristics

We retrospectively reviewed clinical data from the medical records. We selected patients’ characteristics, including sex, age at time of initiation of pembrolizumab and chemotherapy, smoking history, histology, PD-L1 tumor proportion score (TPS) expression, disease stage, treatments received, disease progression and death, or last contact if death had not occurred at the cut-off date. Performance status (PS) was evaluated using the Eastern Cooperative Oncology Group PS scale. The clinical stage was assigned based on computed tomography (CT) of the chest and abdomen, CT or magnetic resonance imaging of the brain, and bone scintigraphy or positron emission tomography. Chest and abdominal CTs were performed every two or three cycles during the treatment to evaluate clinical response. The obtained images were evaluated according to the Response Evaluation Criteria in Solid Tumors (RECIST), version 1.1.

IrAEs were defined as adverse events with a potential immunological basis that required close monitoring or potential intervention with immunosuppressive agents or endocrine therapy. Patient symptoms, physical exploration, and laboratory data were assessed every cycle, and irAEs were based on the physicians’ judgment. We categorized irAEs as colitis, pneumonitis, hepatitis, nephritis, myositis, skin-related irAE, thyroid dysfunction, adrenal insufficiency, and type 1 diabetes mellitus. Fatigue and infusion reactions did not qualify as irAEs in this study. The clinical severity of each irAE was evaluated according to the Common Terminology Criteria for Adverse Events (CTCAE) version 4.0.

PFS was defined as the period between the start of pembrolizumab therapy and progressive disease or death. OS was defined as the period from initiation of treatment to death or the last follow-up. The cut-off date for data collection was May 31, 2021.

### Statistical analysis

Demographic data and characteristics were summarized using median and range for continuous variables and percentages for categorical variables. The chi-square test was used to determine associations between the categorical variables. The Mann–Whitney test evaluated the correlation between continuous variables. Kaplan–Meier survival curves with log-rank tests were used to compare PFS and OS. To reduce the lead time bias, a 12-week landmark analysis was also conducted to evaluate PFS, which included only patients who were progression-free at 90 days after initiation of treatment with pembrolizumab. Univariable and multivariable Cox proportional hazard regression models were used to determine the hazard ratios and confidence intervals. In the univariate analysis, covariates included age (≤ 70 years vs. > 70 years), sex, PS (0/1 vs. ≥ 2), histology (squamous cell lung cancer vs. non-squamous cell lung cancer), postoperative recurrence (yes vs. no), PD-L1 expression (≥ 50% vs. < 50% or unknown), and the presence of irAEs (yes vs. no). Multivariate analysis was performed on variables with *P* < 0.10 in univariate analysis. Potential predictive factors for the development of irAEs were assessed by logistic regression models, using covariates including age, sex, smoking history, PS, histology, PD-L1 expression, postoperative recurrence, and treatment regimen. The inverse probability of treatment weighting (IPTW) with the propensity score was used to adjust for different baseline characteristics between the monotherapy and combination therapy groups, evaluating the difference in frequency of irAEs in both groups.

The IPTW method is used to eliminate bias caused by doctors’ prescription behavior (e.g., whether pembrolizumab monotherapy or pembrolizumab and platinum-based chemotherapy) [9]. Weighting each subject based on propensity score [10], a single numerical value that indicates the probability of patients being exposed to treatment enables adjustment for different baseline characteristics between the monotherapy and combination therapy groups. Let the indicator whether pembrolizumab monotherapy or not be $T$, covariates be ${\varvec{Z}}={({Z}_{1},\dots ,{Z}_{m})}^{{\varvec{T}}}$, and the indicator of whether irAE is $Y$, where T indicates the transport vector. The propensity score of $i$ th subject is ${PS}_{i}=P\left({T}_{i}=1|{{\varvec{Z}}}_{i}={\varvec{z}}\right)(i=1,\dots ,n)$, where $log\left({PS}_{i}/(1-{PS}_{i})\right)={\beta }_{0}+{{\varvec{\beta}}}^{T}{{\varvec{Z}}}_{i} (i=1,\dots ,n)$ and ${{\varvec{\beta}}}^{T}=({\beta }_{1},\dots ,{\beta }_{m})$**.** The weights of $i$-th subject’s outcome is given as ${w}_{i}={T}_{i}/{PS}_{i}+(1-{T}_{i})/{(1-PS}_{i}) (i=1,\dots ,n)$.

Statistical significance was set at *P* < 0.05. All analyses were performed using R version 4.1.0 (R Foundation for Statistical Computing, Vienna, Austria).

## Results

### Characteristics of patients and irAEs profiles

A total of 148 NSCLC patients were included in this study. Baseline clinical characteristics of the patients are shown in Table 1. The median age was 68 years (range 33–80), and 72.3% were men, while 27.7% were women. Most patients were current or former smokers (91.9%). The most common histology was non-squamous cell carcinoma (75.0%). Seventy-four patients were treated with pembrolizumab monotherapy, and 74 with pembrolizumab and platinum-based chemotherapy. The comparison of clinical characteristics between patients who received combination therapy and monotherapy are also shown in Table 1. Age, PS, and PD-L1 TPS were significantly different between the groups. The clinical characteristics of the patients with or without irAEs in each group are shown in Table 2. Statistically significant differences in age and PS were detected between the groups.

**Table 1:** Baseline characteristics of patients treated with combination therapy or monotherapy

	Overall (*n* = 148)	Patients with Combination therapy (*n* = 74) No. (%)	Patients with monotherapy (*n* = 74) No. (%)	*P* value
**Age, median (range), years**	68 (33–88)	68 (33–80)	70.5 (46–88)	< 0.01
**Sex, no. (%)**				
Male	107 (72.3)	54 (73.0)	53 (71.6)	0.85
Female	41 (27.7)	20 (27.0)	21 (28.4)	
**Smoking status, no. (%)**				
Current or Former	136 (91.9)	69 (93.2)	67 (90.5)	0.55
Never	12 (8.1)	5 (6.8)	7 (9.5)	
**Performance status, no. (%)**				
0 or 1	127 (85.8)	69 (93.2)	58 (78.4)	0.01
≥ 2	21 (14.2)	5 (6.8)	16 (21.6)	
**Histological features, no. (%)**				
squamous cell carcinoma	37 (25.0)	16 (21.6)	21 (28.4)	0.34
non squamous cell carcinoma	111 (75.0)	58 (78.4)	53 (71.6)	
**Recurrent after surgery, no. (%)**	54 (36.5)	19 (25.7)	35 (47.3)	0.28
**PD-L1 TPS, no. (%)**				
≥ 50%	87 (58.8)	21 (28.4)	66 (89.2)	< 0.01
< 50% or unknown	61 (41.2)	53 (71.6)	8 (10.8)	

Comparisons were performed using chi-square test and Mann–Whitney U test, as appropriate

*Abbreviations: PD-L1 TPS* Programmed cell death 1-ligand 1 tumor proportion score

**Table 2:** Baseline characteristics of patients with or without irAEs in the combination therapy and monotherapy groups

	Patients with combination therapy, No. (%)			Patients with monotherapy, No. (%)		
	Patients with irAE (*n* = 46)	Patients without irAE (*n* = 28)	*P* value	Patients with irAE (*n* = 40)	Patients without irAE (*n* = 34)	*P* value
**Age, median (range), years**	68 (33–80)	63.5 (40–72)	< 0.01	74 (54–88)	69 (46–85)	0.01
**Sex, no. (%)**						
Male	34 (73.9)	20 (71.4)	0.82	30 (75.0)	23 (53.6)	0.48
Female	12 (26.1)	8 (28.6)		10 (25.0)	11 (32.4)	
**Smoking status, no. (%)**						
Current or Former	44 (95.7)	25 (89.3)	0.29	38 (95.0)	29 (85.3)	0.16
Never	2 (4.3)	3 (10.7)		2 (5.0)	5 (14.7)	
**Performance status, no. (%)**						
0 or 1	41 (89.1)	28 (100)	0.07	35 (87.5)	23 (67.6)	0.04
≥ 2	5 (10.9)	0 (0)		5 (12.5)	11 (32.4)	
**Histological features, no. (%)**						
Non-squamous cell carcinoma	35 (76.1)	23 (82.1)	0.54	29 (72.5)	24 (70.6)	0.86
Squamous cell carcinoma	11 (23.9)	5 (17.9)		11 (14.9)	10 (29.4)	
**Recurrent after surgery,** **no. (%)**	14 (30.4)	5 (17.9)	0.23	10 (25.0)	15 (44.1)	0.08
**PD-L1 TPS, no. (%)**						
≥ 50%	12 (26.1)	9 (32.1)	0.58	37 (92.5)	29 (85.3)	0.32
< 50% or unknown	34 (73.9)	19 (67.9)		3 (7.5)	5 (14.7)	

Comparisons were performed using chi-square test and Mann–Whitney U test, as appropriate

*Abbreviations: irAE* immune-related adverse events, *PD-L1 TPS* Programmed cell death 1-ligand 1 tumor proportion score

The safety profiles are listed in Table 3. In patients treated with pembrolizumab plus chemotherapy, 46 patients (62.2%) experienced irAEs of any grade, including 14 patients (18.9%) who experienced two or more irAEs. Seventeen patients (23.0%) exhibited irAEs of grade 3 or higher. Sixteen patients required systemic steroid therapy for the treatment of irAEs. Sixteen patients (21.6%) discontinued treatment with pembrolizumab and chemotherapy due to irAEs.

**Table 3:** The safety profiles of irAEs in the combination therapy and monotherapy groups

	Patients with combination therapy, No. (%)						Patients with monotherapy, No. (%)					
	Total (*n* = 74)	Grade 1–2	Grade 3–4	Systemic Steroid Therapy	Discontinuation of chemotherapy due to irAE	Median onset time, median (min–max), weeks	Total (*n* = 74)	Grade 1–2	Grade 3–4	Systemic Steroid Therapy	Discontinuation of chemotherapy due to irAE	Median onset time, median (min–max), weeks
**All irAEs**	46 (62.2)	29 (39.2)	17 (23.0)	16	16	9.1 (1.0–58.3)	41 (55.4)	32 (43.2)	9 (12.2)	15	18	9.0 (0.0–79.1)
One irAE	32 (43.2)						24 (32.4)					
Two or more	14 (18.9)						16 (21.6)					
**Each irAEs**												
**Gastrointestinal**	4 (5.4)	2 (2.7)	2 (2.7)	2	2	17.4 (1.6–47.7)	3 (4.1)	-	3 (4.1)	1	2	1.4 (0.7–4.9)
**Pneumonitis**	15 (20.3)	11 (14.9)	4 (5.4)	7	6	18.0 (1.9–46.1)	13 (17.6)	10 (13.5)	3 (4.1)	7	9	12.1 (0.6–71.1)
**Hepatitis**	2 (2.8)	1 (1.4)	1 (1.4)	1	1	3.4 (1.4–5.4)	-	-	-			-
**Myositis**	5 (6.8)	3 (4.1)	2 (2.8)	1	2	4.0 (1.0–53.4)	4 (5.4)	3 (4.1)	1 (1.4)	2	3	7.1 (6.0–55.0)
**Nephritis**	1 (1.4)	-	1 (1.4)	1	1	2.0	1 (1.4)	1 (1.4)	-	1	1	15.3
**Meningitis or Encephalitis**	-	-	-	-	-	-	2 (2.7)	-	2 (2.7)	2	2	2.0 (1.3–6.7)
**Skin**	21 (28.4)	20 (27.0)	1 (1.4)	0	0	2.4 (1.0–58.3)	26 (35.1)	25 (33.8)	1 (1.4)	1	0	9.6 (0–77.1)
**Endocrine**												
Thyroid dysfunction	9 (12.2)	8 (10.8)	1 (1.4)		1	6.9 (3.0–26.6)	4 (5.4)	3 (4.1)	1 (1.4)	0	0	18.9 (3.4–48.1)
Adrenal insufficiency	4 (5.4)	-	4 (5.4)	4	2	26.9 (12.4–48.0)	1 (1.4)	1 (1.4)	1 (1.4)	1	1	42.9
Type 1 DM	1 (1.4)	-	1 (1.4)		1	21.9	-	-	-			-

*Abbreviations: irAE* immune-related adverse events, *DM* Diabetes mellitus

Conversely, in patients receiving pembrolizumab monotherapy, 41 patients (55.4%) experienced irAEs of any grade. Nine patients had irAEs of grade 3 or higher. Fifteen patients required systemic steroid therapy, and 18 patients discontinued pembrolizumab. In both the groups, the most common irAEs were skin toxicity and pneumonitis. In addition, the most frequent irAE associated with treatment discontinuation was pneumonitis. The median time to the onset of irAEs is shown in Table 3. In patients who received combination therapy, the median time of onset of any grade irAE was 9.1 weeks, whereas that in patients who received monotherapy was 9.0 weeks. The median onset time of each irAE was also determined; however, there were no differences between the two groups.

Among all irAEs, 26 patients (17.6%) experienced irAEs of grade 3 or higher. In particular, the incidence of pneumonitis was 5.4% and 4.1% in patients who received combination therapy and monotherapy, respectively. In addition, 8 patients (5.4%) developed endocrine toxicities. Four out of 5 patients who stopped their immunotherapy, actually suspended their immunotherapy based on the physicians’ judgement because their disease was under control. Three patients with severe irAEs received rechallenge immunotherapy and continued ICIs, and their disease was under control at the cut-off date.

### Association between irAEs and efficacy

The median follow-up duration for all patients was 14.9 months (range, 0.8–53.3), 13.2 months (range, 0.8–28.3) for combination therapy and 17.4 months (range, 0.0–53.3) for monotherapy. The median PFS and OS of the study population were 5.4 months (range, 0.1–39.8) and 12.6 months (range, 0–61.3), respectively.

**Fig. 1:**
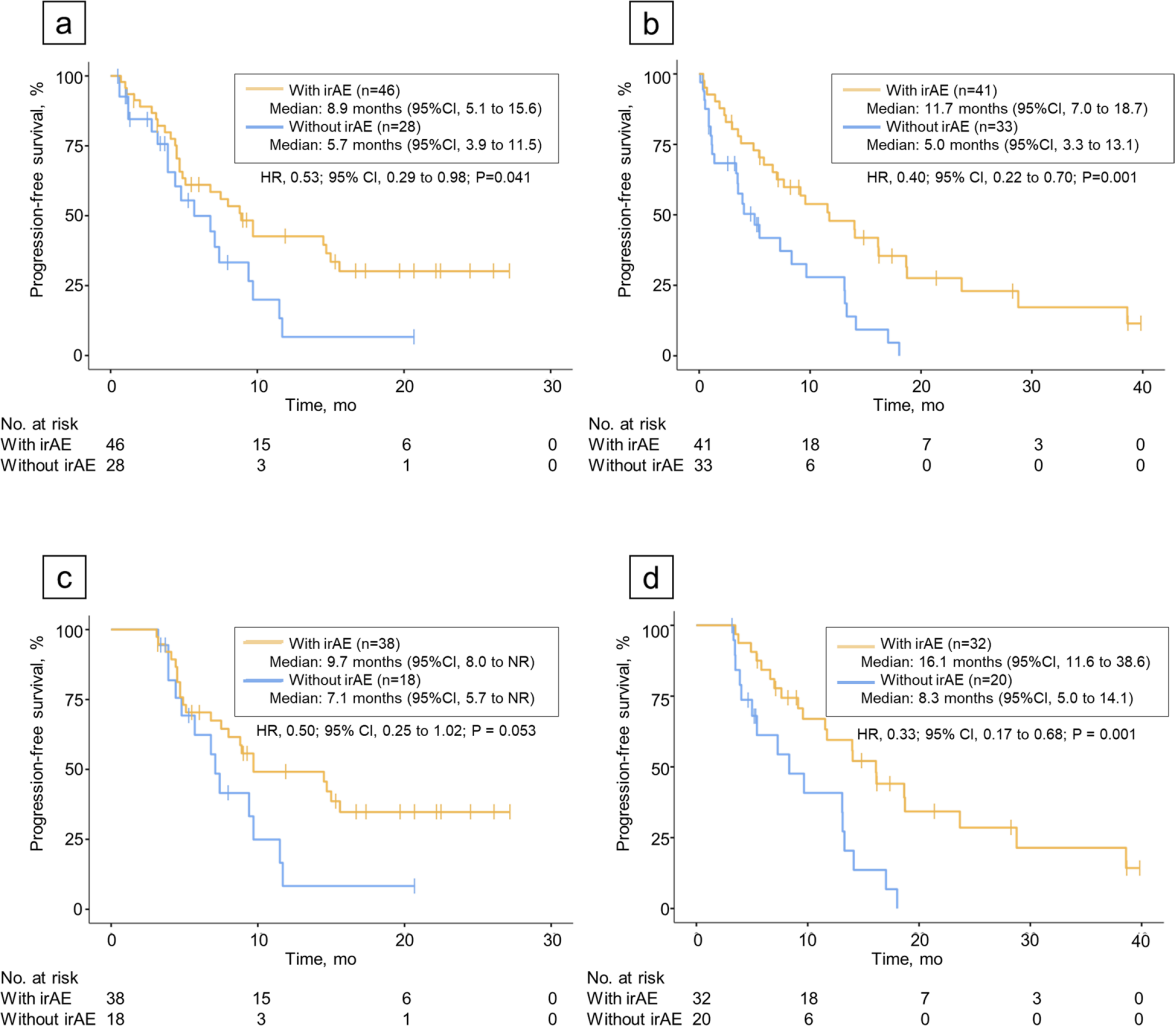
IrAEs and efficacy. Kaplan–Meier curves for progression-free survival in patients treated with pembrolizumab and chemotherapy (**a**) and those treated with pembrolizumab monotherapy (**b**). Kaplan–Meier curves with 12-week landmark analysis for progression-free survival in patients treated with pembrolizumab and chemotherapy (**c**) and those treated with pembrolizumab monotherapy (**d**)

In patients treated with combination therapy, the median PFS was 8.9 months (95% CI, 5.1–15.6) in patients with irAE vs. 5.7 months (95% CI, 3.9–11.5) in patients without irAE. Thus, the development of irAEs was significantly associated with longer PFS (HR, 0.53; 95% CI, 0.29–0.98; *P* = 0.041; Fig. 1a). Similar results were observed in patients treated with pembrolizumab monotherapy. The median PFS among patients with and without irAEs was 11.7 months (95% CI, 7.0–18.7) and 5.0 months (95% CI, 3.3–18.7), respectively, indicating statistically longer PFS in the former group (HR, 0.40; 95% CI, 0.22–0.70; *P* = 0.001; Fig. 1b). In patents with irAEs of grade 3 or higher, PFS was significantly longer compared to that in patients without irAEs (supplementary figure S2).

In the 12-week landmark analysis for PFS, 18 patients treated with combination therapy and 22 who received monotherapy were excluded because of disease progression within 90 days of pembrolizumab treatment initiation. Patients treated with combination therapy showed a trend toward a longer PFS if an irAE was documented [9.7 months vs. 7.1 months, (HR, 0.50; 95% CI, 0.25–1.02; *P* = 0.053); Fig. 1c]. Within the monotherapy patient group, the median PFS was 16.1 months (95% CI, 11.6–38.6) for patients with irAEs compared to 8.3 months (95% CI, 5.0–14.1) for patients without irAEs (HR, 0.33; 95% CI, 0.17–0.68; *P* = 0.001; Fig. 1d). In addition to landmark analysis, we also performed time-varying Cox model analysis from 90 days after treatment initiation (Supplemental Table S1). “The presence of irAE” in patients with monotherapy showed statistical significance in the multivariate analysis (HR 0.42; 95% CI, 0.21–0.85), whereas no statistical significance was observed in patients with combination therapy.

The data recorded on OS turned out inconclusive in patients treated with the combination therapy (Supplemental Fig. 1a). However, in patients with monotherapy, the development of an irAE was significantly associated with improved OS {40.4 months [95% CI, 38.6–not reached (NR)] vs. 21.8 months (95% CI, 11.0–NR); HR, 0.40; 95% CI, 0.19–0.86; *P* = 0.015; Supplemental 1b}.

In a multivariable PFS analysis, the presence of irAEs was significantly associated with longer PFS in both patient groups; with combination therapy (HR, 0.48; 95% CI, 0.26–0.89; *P* = 0.019) and monotherapy (HR, 0.38; 95% CI, 0.21–0.68; *P* < 0.01; Table 4).

**Table 4:** Univariable and multivariable analysis of covariates for progression-free survival in the combination therapy and monotherapy groups

	Patients with combination therapy						Patients with monotherapy					
	Univariate analysis			Multivariate analysis			Univariate analysis			Multivariate analysis		
	HR	95% CI	*P* value	HR	95% CI	*P* value	HR	95% CI	*P* value	HR	95% CI	*P* value
**Age** (≤ 70y vs. > 70 y)	1.09	0.59–2.02	0.79				1.11	0.65–1.91	0.71			
**Sex** (Male vs. Female)	0.95	0.49–1.84	0.88				1.13	0.61–2.08	0.70			
**Performance status** (0–1 vs. ≥ 2)	0.40	0.05–2.88	0.36				2.75	1.39–5.45	0.00	0.40	0.17–0.93	0.03
**Histology** (non-SQ vs. SQ)	0.98	0.49–1.98	0.96				2.27	1.19–4.32	0.01	1.62	0.78–3.38	0.20
**Postoperative recurrence** (Yes vs. No)	0.59	0.30–1.17	0.13				1.23	0.71–2.12	0.46			
**PD-L1 TPS** (≥ 50% vs. < 50% or unknown)	1.88	0.90–3.89	0.09	0.47	0.23–0.99	0.047	0.99	0.39–2.49	0.98			
**The presence of irAE** (Yes vs. No)	0.53	0.29–0.98	0.04	0.48	0.26–0.89	0.019	0.40	0.22–0.70	< 0.01	0.38	0.21–0.68	< 0.01

*Abbreviations: HR* Hazard ratio, *CI* Confidence interval, *SQ* Squamous cell carcinoma, *PD-L1 TPS* Programmed cell death 1- ligand 1 tumor proportion score, *irAE* immune-related adverse events

### The predictors of irAE development

In a logistic regression analysis, the development of irAEs showed a tendency of being higher in patients treated with combination therapy than in those treated with monotherapy(OR 0.41; 95% CI, 0.14–1.14; *P* = 0.09). In addition, age (> 70 years) was a significant risk factor for the development of irAEs (OR 5.4; 95% CI, 2.27–13.90; *P* < 0.01; Table 5). However, since this logistic regression analysis appeared to involve many covariates, we subsequently performed the IPTW method to compare the frequencies of irAEs between the monotherapy and combination therapy patient groups. The IPTW-adjusted analysis showed that the frequency of irAEs was significantly higher in patients treated with combination therapy than in those treated with monotherapy (OR 0.56; 95% CI, 0.34–0.91, *P* = 0.019; Table 5).

**Table 5:** Logistic regression analysis and inverse probability of treatment weighting-adjusted analysis for irAEs in all patients

	Without IPTW						With IPTW		
	Univariate analysis			Multivariate analysis					
	OR	95% CI	*P* value	OR	95% CI	*P* value	OR	95% CI	*P* value
**Age** (≤ 70y vs. > 70 y)	3.77	1.82–8.20	< 0.01	5.40	2.27–13.90	< 0.01			
**Sex** (Male vs. Female)	0.78	0.37–1.62	0.50	0.93	0.39–2.25	0.87			
**Smoking histology** (Never vs. Past/Current)	3.04	0.90–11.96	0.09	3.62	0.87–17.52	0.09			
**Performance status** (0–1 vs. ≥ 2)	0.61	0.24–1.56	0.30	1.18	0.87–17.52	0.78			
**Histology** (Non-SQ vs. SQ)	1.11	0.51–2.45	0.80	0.92	0.38–2.25	0.85			
**PD-L1 TPS** (≥ 50% vs. < 50% or Unknown)	1.20	0.61–2.35	0.60	1.28	0.50–3.27	0.65			
**Postoperative recurrence** (No vs. Yes)	0.81	0.40–1.67	0.57	0.79	0.34–1.82	0.58			
**Treatment regimen** (Combination therapy vs. Monotherapy)	0.72	0.37–1.38	0.32	0.41	0.14–1.14	0.09	0.56	0.34–0.91	0.019

*Abbreviations: IPTW* Inverse probability of treatment weighting, *OR* Odds ratio, *CI* Confidence interval, *SQ* Squamous cell carcinoma, *PD-L1 TPS* Programmed cell death 1-ligand 1 tumor proportion score, *irAE* immune-related adverse

## Discussion

Our study revealed the frequencies of irAEs as well as the association between irAEs and better efficacy in patients treated with pembrolizumab with or without chemotherapy in a real-world setting. To the best of our knowledge, this is the first report describing a correlation between the development of irAEs and a favorable efficacy in advanced NSCLC patients treated with pembrolizumab plus chemotherapy. We also showed that pembrolizumab plus platinum-based chemotherapy entails the development of more frequent irAEs than pembrolizumab monotherapy by using IPTW methods.

In our study, the rate of irAEs, especially pneumonitis, was higher compared to that in prior clinical trials. The frequency of irAEs has been reported to be higher in the real-world data than in clinical trials, and in patients receiving immunotherapy in the first-line treatment than in the second-line therapy or beyond [11]. In addition, racial differences may be implicated in pneumonia. More patients were diagnosed with pneumonitis in Japanese prospective clinical trials than those in the western countries. For example, Fujimoto et al. reported an incidence of 12.4% for all-grade pneumonitis and 3.3% for grade ≥ 3 pneumonitis in a retrospective cohort study in Japan, which are higher rates of pneumonitis than those reported in western countries [12].

ICIs work by blocking negative regulators of T-cell activation that exist both on tumor and immune cells [13]. IrAEs are believed to arise from the general immunologic enhancement of T-cells, although the precise mechanisms of irAEs are not fully understood [13, 14]. The relationship between the development of irAEs and a favorable prognosis has been reported. Concerning PD-1 antibody monotherapy, several studies have recently shown that patients who experienced irAEs showed significant improvements regarding response rate, PFS, and OS than patients without irAEs [8, 11, 15–17]. These outcomes were comparable to our results in patients treated with pembrolizumab monotherapy. In addition, the present study is the first to show that the presence of irAEs was also associated with longer PFS in patients treated with pembrolizumab and platinum-based chemotherapy. The multivariable analysis revealed that irAEs were significantly associated with prolonged PFS. The results of time-varying Cox model analysis did not contradict the results shown in the multivariable analysis since the HRs of “the presence of irAE” are not statistically significant. Although the follow-up period was not long enough to assess OS, this study identified that the development of irAEs was associated with the favorable efficacy in patients treated with pembrolizumab with or without chemotherapy.

We used IPTW methods to balance baseline patient characteristics between the monotherapy and combination therapy groups and compared the frequency of irAEs between both groups. In the adjusted IPTW analysis, combination therapy was a potential risk factor for the development of irAEs. One possible explanation is the immunostimulatory effect of chemotherapy, which might entail an increased occurrence of irAEs. Conventional chemotherapies have been reported to elicit anticancer immune responses where the direct effects on cancer cells and the indirect effects on various immune cell subsets can be distinguished [18]. Such immunostimulatory effects of chemotherapeutic agents may explain the increase in the frequency of irAEs when used in combination with ICIs [18]. Another hypothesis is that additional chemotherapy may lead to an overestimation of irAEs. It is challenging to distinguish irAEs from the side effects of anti-cytotoxic agents in clinical practice. In particular, pneumonitis could develop due to both ICIs and cytotoxic anticancer agents, affecting the frequency of irAEs in the combination therapy group. However, in our study, all irAE classifications were based on the attending physician’s judgment; thus, these still remain informative data for clinical setting.

Notably, age (> 70 years) was also a potential risk factor for the development of irAEs. On one hand, previous studies have shown that irAEs were more frequent in elderly patients [19]. Aging of the immune system, age-related complications, and reduced functional reserves may be possible causes [20]. On the other hand, some reports showed no significant difference in the development of irAEs between older and younger individuals [21, 22]. Although there is no consensus on the relationship between age and the development of irAEs, further studies in elderly patients are needed to clarify the role of age in the development of irAEs; caution is advised when treating elderly patients.

There were some limitations to our study. First, this was a retrospective study in a single center with limited sample size; irAEs could have been inevitably underreported or all treatments for irAEs were determined by physicians due to the study's retrospective nature. Therefore, prospective or additional cohort validation is needed to verify our findings in the future. Second, the follow-up period was not sufficient to evaluate long-term survival. In contrast, our study's strengths consist of assessing irAEs during first-line therapy rather than analyzing them during both first-line and later-line therapy. Furthermore, we focused on one specific immune checkpoint inhibitor, pembrolizumab, and compared monotherapy with combination therapy given simultaneously, minimizing the effects of additional confounding factors. Although further investigation is required, our data showing irAE of combination therapy and monotherapy would be informative and clinically meaningful.

## Conclusion

Our study showed that the presence of irAEs is associated with longer PFS in NSCLC patients treated with pembrolizumab plus chemotherapy, and adding chemotherapy to pembrolizumab significantly increased the prevalence of irAEs.

## Supplementary Information

**Additional file 1: Supplementary Table S1.** Univariable and multivariable time-varying Cox model analysis of covariates for progression-free survival in the combination therapy and monotherapy groups.

**Additional file 2: Supplementary figure S1.** IrAEs and survival. Kaplan–Meier curves for overall survival in patients treated with pembrolizumab and chemotherapy (a) and those treated with pembrolizumab monotherapy (b).

**Additional file 3: Supplementary figure S2.** Severe irAEs and efficacy. Kaplan–Meier curves for progression-free survival in patients with irAEs of grade 3 or higher versus patients without irAEs.

## Data Availability

The datasets supporting the conclusions of this article are included within the article.

## Abbreviations

NSCLC: Non-small cell lung cancer
PD-1: Programmed cell death 1
PD-L1: Programmed cell death ligand 1
PFS: Progression-free survival
OS: Overall survival
ICIs: Immune checkpoint inhibitors
irAEs: Immune-related adverse events
TPS: Tumor proportion score
PS: Performance status
CT: Computed tomography
IPTW: Inverse probability of treatment weighting
NR: Not reached
