# Supplementary material for: Association between the efficacy and immune-related adverse events of pembrolizumab and chemotherapy in non-small cell lung cancer patients: a retrospective study
Source: BMC Cancer. 2022 Oct 6;22:1047. doi: 10.1186/s12885-022-10133-1 (PMC9535983; doi:10.1186/s12885-022-10133-1)
Supplement: Supplementary file 1 — Additional file 1: Supplementary Table S1. Univariable and multivariable time-varying Cox model analysis of covariates for progression-free survival in the combination therapy and monotherapy groups. [file 12885_2022_10133_MOESM1_ESM.docx]

**Supplementary Table S1:** Univariable and multivariable time-varying Cox model analysis of covariates for progression-free survival in the combination therapy and monotherapy groups

|  | Patients with combination therapy | | | | | | |  | Patients with monotherapy | | | | | | |
| --- | --- | --- | --- | --- | --- | --- | --- | --- | --- | --- | --- | --- | --- | --- | --- |
|  | Univariate analysis | | | | Multivariate analysis | | |  | Univariate analysis | | | | Multivariate analysis | | |
|  | HR | 95% CI | *P* value | HR | | 95% CI | *P* value |  | HR | 95% CI | *P* value | HR | | 95% CI | *P* value |
| **Age** (≤70y vs. >70 y) |  |  |  |  | |  |  |  |  |  |  |  | |  |  |
| **Sex** (Male vs. Female) |  |  |  |  | |  |  |  |  |  |  |  | |  |  |
| **Performance status** (0-1 vs. ≥2) |  |  |  |  | |  |  |  |  |  |  | 1.18 | | 0.51-2.71 | 0.698 |
| **Histology** (non-SQ vs. SQ) |  |  |  |  | |  |  |  |  |  |  | 6.13 | | 2.42-15.55 | <0.01 |
| **Postoperative recurrence** (Yes vs. No) |  |  |  |  | |  |  |  |  |  |  |  | |  |  |
| **PD-L1 TPS** (≥50% vs. <50% or unknown) |  |  |  | 1.98 | | 0.85-4.62 | 0.395 |  |  |  |  |  | |  |  |
| **The presence of irAE** (Yes vs. No; Time-Varying) | 0.84 | 0.43-1.65 | 0.610 | 0.74 | | 0.37-1.49 | 0.385 |  | 0.53 | 0.28-0.99 | 0.046 | 0.42 | | 0.21-0.85 | 0.017 |

Abbreviations: HR, hazard ratio; CI, confidence interval; SQ, squamous cell carcinoma; PD-L1 TPS, programmed cell death 1- ligand 1 tumor proportion score; irAE, immune-related adverse events.
