# Supplementary figures and images for: Association between the efficacy and immune-related adverse events of pembrolizumab and chemotherapy in non-small cell lung cancer patients: a retrospective study
Source: BMC Cancer. 2022 Oct 6;22:1047. doi: 10.1186/s12885-022-10133-1 (PMC9535983; doi:10.1186/s12885-022-10133-1)

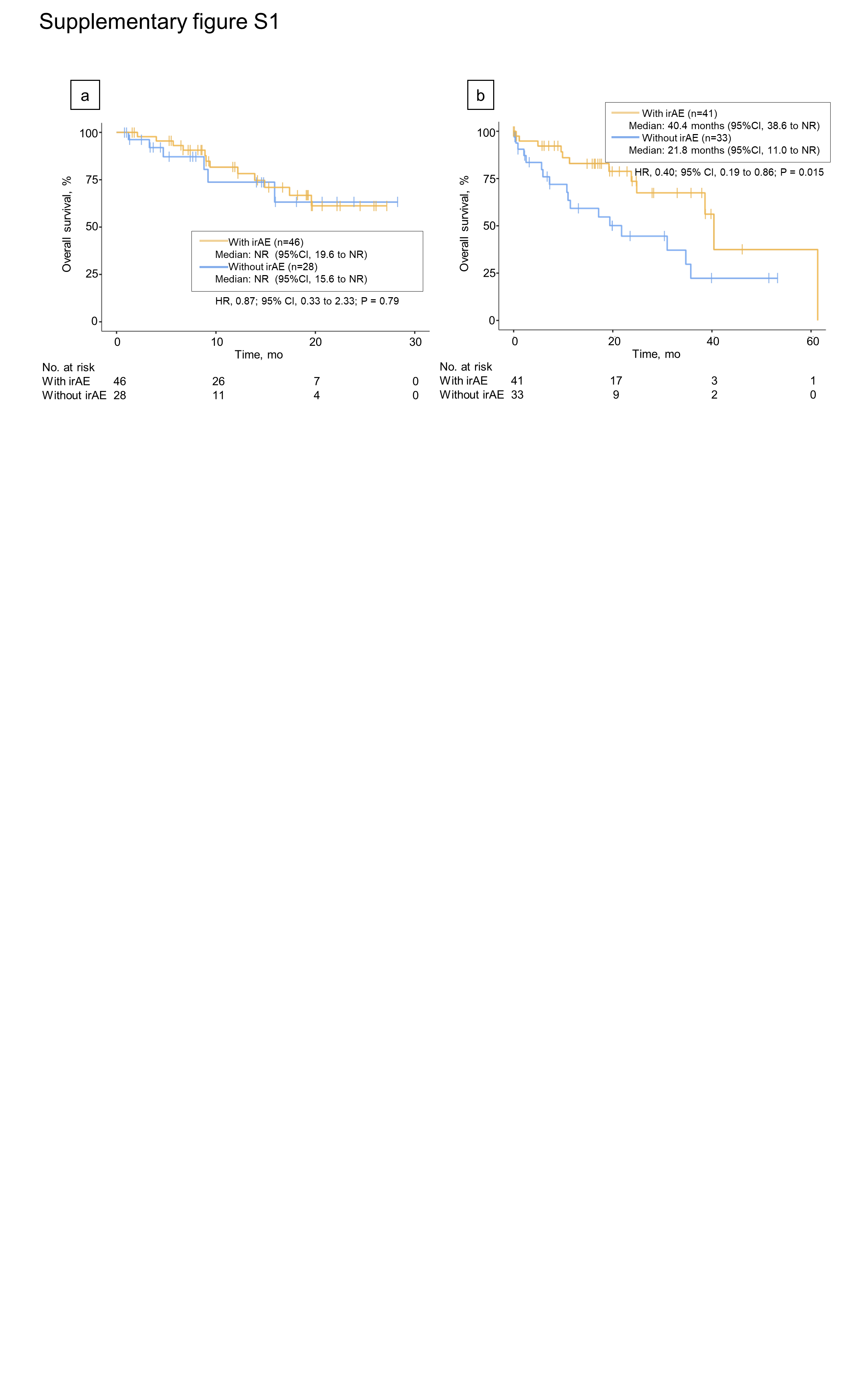

Supplement: Supplementary file 2 — Additional file 2: Supplementary figure S1. IrAEs and survival. Kaplan–Meier curves for overall survival in patients treated with pembrolizumab and chemotherapy (a) and those treated with pembrolizumab monotherapy (b). [file 12885_2022_10133_MOESM2_ESM.tif]

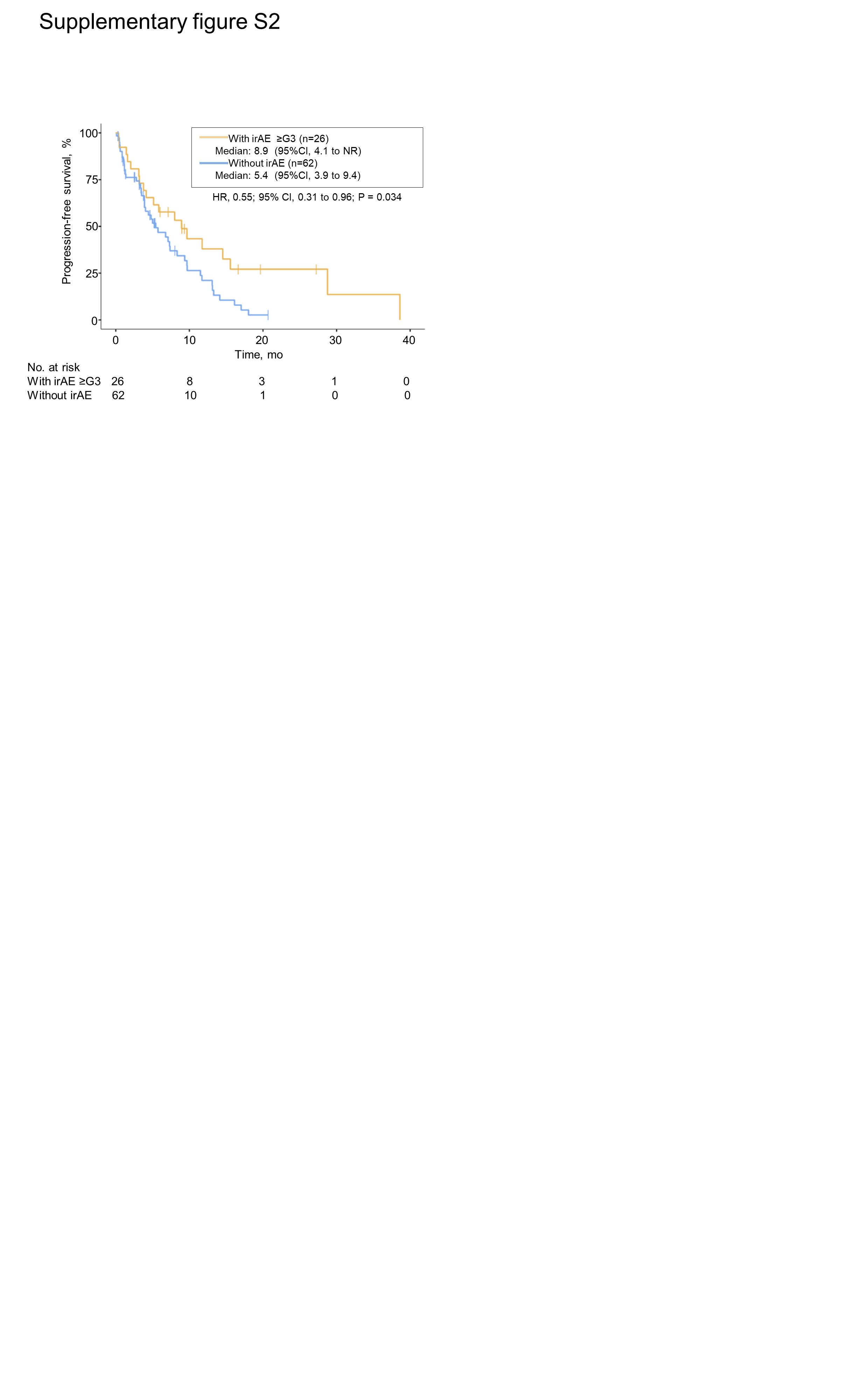

Supplement: Supplementary file 3 — Additional file 3: Supplementary figure S2. Severe irAEs and efficacy. Kaplan–Meier curves for progression-free survival in patients with irAEs of grade 3 or higher versus patients without irAEs. [file 12885_2022_10133_MOESM3_ESM.tif]
